# Glyphosate Interference in Follicular Organization in the Wall Lizard *Podarcis siculus*

**DOI:** 10.3390/ijms24087363

**Published:** 2023-04-17

**Authors:** Luigi Rosati, Teresa Chianese, Vincenza De Gregorio, Mariailaria Verderame, Anja Raggio, Chiara Maria Motta, Rosaria Scudiero

**Affiliations:** 1 Department of Biology, University Federico II, Via Cintia 21, 80126 Napoli, Italy; 2 Department of Human, Philosophic and Education Sciences (DISUFF), University of Salerno, 84084 Fisciano, Italy

**Keywords:** oogenesis, reptiles, reproductive toxicology, estrogen disruptors

## Abstract

Glyphosate (Gly) is a broad-spectrum herbicide widely used thanks to its high efficiency and low toxicity. However, evidence exists of its toxic effects on non-target organisms. Among these, the animals inhabiting agricultural fields are particularly threatened. Recent studies demonstrated that exposure to Gly markedly affected the morphophysiology of the liver and testis of the Italian field lizard *Podarcis siculus*. The present study aimed to investigate the effects of the herbicide on the female reproductive system of this lizard in order to have a full picture of Gly-induced reproductive impairment. The animals were exposed to 0.05 and 0.5 μg/kg of pure Gly by gavage for 3 weeks. The results demonstrated that Gly, at both doses tested, profoundly interfered with ovarian function. It induced germ cells’ recruitment and altered follicular anatomy by anticipating apoptotic regression of the pyriform cells. It also induced thecal fibrosis and affected oocyte cytoplasm and zona pellucida organizations. At the functional levels, Gly stimulated the synthesis of estrogen receptors, suggesting a serious endocrine-disrupting effect. Overall, the follicular alterations, combined with those found at the level of the seminiferous tubules in males, suggest serious damage to the reproductive fitness of these non-target organisms, which over time could lead to a decline in survival.

## 1. Introduction

Modern intensive practices in agriculture provide for a wide use of herbicides and, among these, of glyphosate (Gly). In 2020, the global production of this synthetic molecule was around 700,000 tons [1], and the increasing presence of Gly in the environment has raised concerns about human safety. Gly has been found in human urine following its ingestion through contaminated vegetables [2,3]. Experimental exposure of mammals to Gly had mutagenic and carcinogenic effects [4,5,6], causing neurotoxicity, hepatotoxicity, and disruption of the reproductive axis [1,7]. As a consequence, fertility is greatly reduced by Gly; implantation failures [8] and teratogenesis [9] have been detected, as well as interferences with both spermatogenesis and oogenesis [10]. In male rodents, abnormalities in sperm viability and concentration [11,12,13], in testicular morphology [14], and in aromatase and steroidogenic acute regulatory protein (StAR) expression [15,16] have been reported. In female mammals, exposure to Gly and Gly-based herbicides (GBHs) induced ovarian histopathological alterations and, in particular, arrest of oocyte maturation, increased follicular atresia and interstitial fibrosis, with alterations of serum concentrations of sex hormones and gonadotrophins [1,2,7,17].

Regarding wild species that may be exposed to glyphosate in their natural environment, studies have mainly focused on aquatic organisms, as they can easily come into contact with wastewater from the irrigation of cultivated fields. Glyphosate is in fact highly soluble in water [18]. In both invertebrates and vertebrates, in vivo exposure to Gly or GBH induces damage similar to that observed in mammals, especially in the reproductive system [19,20]. In zebrafish, Gly reduced fecundity and increased early-stage embryo mortalities and premature hatching. In both sexes, mechanisms of reproductive toxicity include disruption of the steroidogenic biosynthetic pathway and oxidative stress [19].

Recently, the effects of Gly were determined on the wall lizard *Podarcis siculus*. Inhabiting areas intended for agriculture, lizards can easily be exposed to this herbicide, through contaminated water and food (insects, earthworms, and vegetables). *P. siculus* is a good bioindicator in ecotoxicology; several studies have demonstrated that this lizard can be contaminated in its natural habitat by herbicides, pesticides, and manure, which modify its health status and give rise to measurable biological responses [21,22,23].

The severe liver fibrosis, the activation of the antioxidant defence machinery, and the xenoestrogenic effects of Gly in *P. siculus* [24] prompted a broadening of the investigations into reproductive toxicity. In males, Gly altered testicular morphology, affected spermatogenesis, and changed the localization of estrogen receptors in germ cells [25].

The aim of this work was to expand the study to the female reproductive system of *P. siculus*. For this purpose, the structural and functional organization of the ovarian follicles following oral exposure to the herbicide was investigated. In particular, the effects on the number of germ cells and follicles were determined, while epithelial/thecal alterations were detected by cytological investigations. Immunocytochemistry detected alterations (1) in PCNA expression, indicative of alterations in proliferation rate [26]; (2) in E-cadherin localization, indicative of a loss of contact between follicle cells [27]; and (3) in estradiol alpha and beta receptors, indicative of an interference with the endocrine axis [5,24].

*P. siculus* ovaries are characterized by a cluster structure, with prefollicular cells located in two germinal beds and follicles showing a complex epithelium, functional to the maturation and growth of the oocyte. The primary follicles are surrounded by a monolayer of small stem cells, which divide rapidly into two cells, one that maintains a stem function and remains close to the external connective theca and another that contacts the oocyte, forming an intercellular bridge. Then, follicle cells markedly increase in size and change shape, becoming pyriform cells [28]. The pyriform cells are nurse cells that degenerate via apoptosis before vitellogenesis starts [29]. At this stage, small stem cells form the steroidogenetic epithelium of the vitellogenic follicles [30]. In *P. siculus*, reproduction occurs with two or three ovulatory waves in spring/summer, followed by a summer resting period and a fall recrudescence, in which ovarian functions are partly resumed but quite soon arrested by the beginning of the winter stasis [31].

The results demonstrated that glyphosate profoundly interferes with ovarian structure and function. It anticipates germ cells’ recruitment and alters follicular anatomy by anticipating apoptotic regression of the pyriform cells. It also induces thecal fibrosis and affects the oocyte cytoplasm and the organization of the zona pellucida. At the functional level, Gly stimulates estrogen receptors’ synthesis, suggesting a severe endocrine-disrupting effect.

## 2. Results

### 2.1. Effects of Glyphosate on Ovarian Anatomy

**Figure 1:**
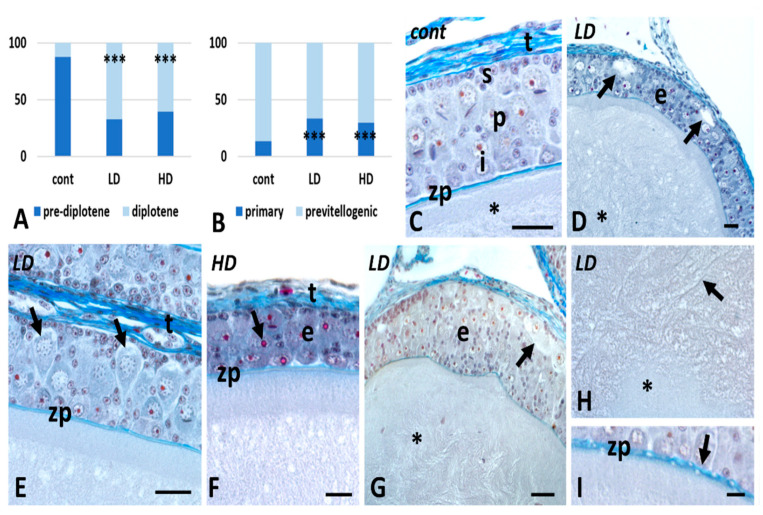
Effects of glyphosate (low LD and high HD doses) on follicle anatomy. Significant increase in prefollicular diplotene oocytes (**A**) and primary follicles (**B**) (***, *p* < 0.001). (**C**) Oocyte with regular cytoplasm (*), zona pellucida (zp), and polymorphic epithelium composed of small (s), pyriform (p), and intermediate (i) cells. Connective theca (t). (**D**) Apoptotic follicle cells (arrows) in the epithelium (e) and disorganized oocyte cytoplasm (*). (**E**) Detail of several early apoptotic cells (arrows). Notice the loss of contact with adjacent cells. Group of thecal cells (t). (**F**) Detail of a patch of condensed follicular epithelium (e). Cells still have a nucleolus (arrow) and the theca is compacted (t). (**G**) Thickened epithelium (e) with several apoptotic cells (arrow). Altered oocyte cytoplasm (*). (**H**) Detail of the oocyte cytoplasm with homogeneous (*) and grossly fibrous (arrow) areas. (**I**) Zona pellucida with discontinuities (arrow). Galgano’s trichrome staining; scale bars: (**C**–**G**) 25 µm; (**H**,**I**) 10 µm.

The ovary of control animals contains an average of 458 ± 24 standard deviation (SD) prefollicular oogonia and oocytes and 13.2 ± 0.75 SD previtellogenic follicles, ranging from 80 to about 1400 microns in diameter (Figure 1A,B). Early diplotene oocytes represent about 12.3% of total prefollicular germ cells; primary follicles are 13.4% of total follicles. After exposure to glyphosate, the number of early diplotene oocytes increases dramatically, reaching 32.6 and 39.2% of the entire population. Only occasional zygotene oocytes are found. The total number of prefollicular oocytes also increases as several new primary follicles are recruited (33.3% and 29.4% of the total; Figure 1A,B).

In sections, control oocytes show a homogenous, fibro-granular cytoplasm delimited by a continuous zona pellucida and a multi-layered epithelium. This is typically polymorphic, with large pyriform cells occupying most of the thickness, small cells located immediately under the connective theca, and intermediate cells close to the zona pellucida (Figure 1C).

In glyphosate-treated follicles, no matter the dose, the epithelium shows several late (Figure 1D) or early (Figure 1E) apoptotic pyriforms, recognizable for the disorganized cytoplasm and the loss of contact with the adjacent cells. In addition, in many oocytes, there are large areas in which the epithelium is very compact, and cells show a very dense but not disorganized cytoplasm (Figure 1F). Patches of thickened epithelium are also present (Figure 1G). In all exposed samples, the oocyte cytoplasm is disorganized, with clearly distinguishable homogeneous and fibrous areas (Figure 1G,H); the zona pellucida is irregular and often interrupted (Figure 1I).

### 2.2. Effects of Glyphosate on Collagen Deposition

**Figure 2:**
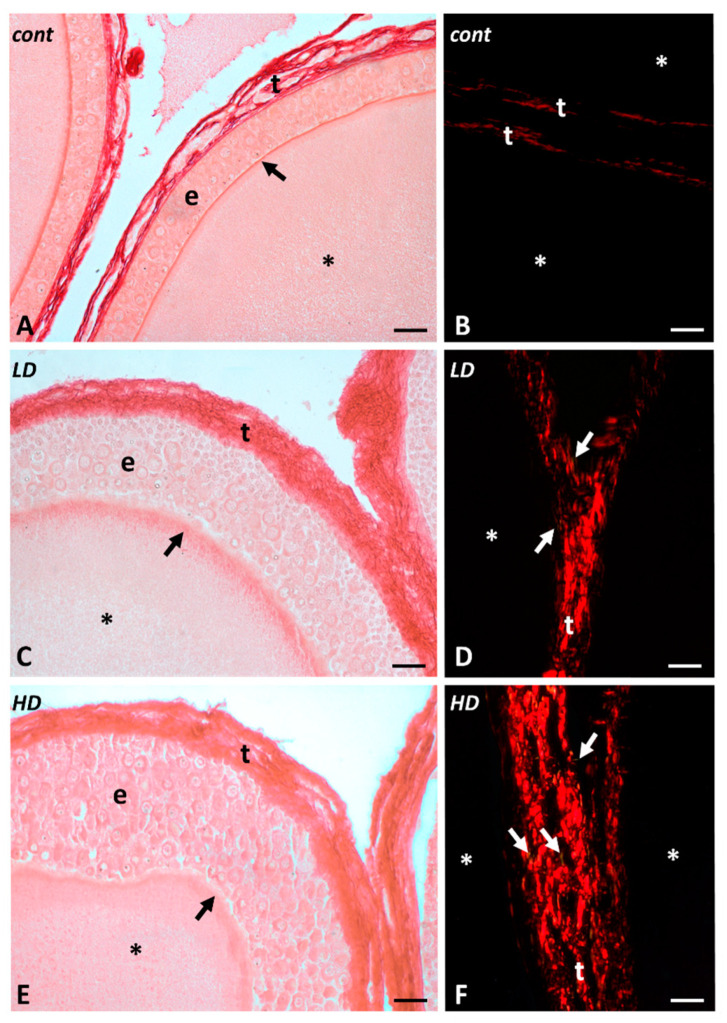
Collagen localization in *P. siculus* follicles exposed to glyphosate (low LD and high HD doses). Picrosirius red staining observed under bright-field light (left panels) or polarized light (right panels). (**A**,**C**,**E**) Progressive thickening and compaction of the connective theca (t). Epithelium (e), zona pellucida (arrows), and oocyte cytoplasm (*). (**B**,**D**,**F**) Progressive increase in mature (red) and immature (green, arrows) fibers in connective theca. Scale bars 20 μm.

Picrosirius red (PSR) staining shows a dose-dependent increase in matrix deposition in both low- and high-dose glyphosate-treated samples (Figure 2A,C,E).

**Figure 3:**
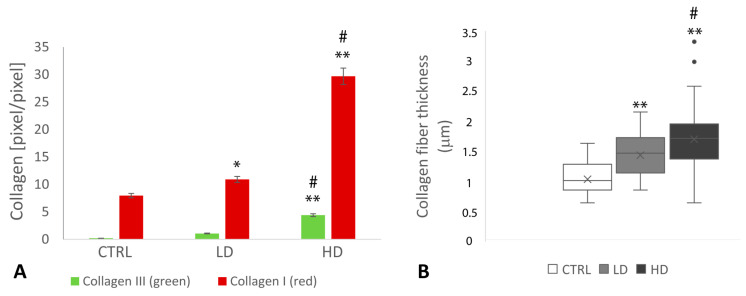
(**A**) Quantitative examination of polarized light images of PSR-stained micrographs. Comparison of mature and immature collagen deposition in high dose (HD) and low dose (LD) vs. control (CTRL) (* *p* < 0.05; ** *p* < 0.001); HD vs. LD (# *p* < 0.001). (**B**) Mean collagen fiber diameter in HD- and LD-treated samples compared with the control group. HD and LD vs. CTRL (** *p* < 0.001); HD vs. LD (# *p* < 0.001).

PSR staining observed under polarized light allows recognition of thin immature collagen fibers (collagen III, green stained) from the thick mature collagen fibers (collagen I, red stained). The results indicate that immature and mature collagens increased dose-dependently in treated samples with respect to controls (Figure 2B,D,F, respectively, and Figure 3A). Newly deposited immature collagen fibers are also found in both low- and high-dose-treated samples, suggesting the presence of poorly packed collagen fibers in the interstitial area of the theca (Figure 3A). Of note, quantitative analysis of collagen fibers’ diameter reveals that the collagen network of high-dose-treated samples is densely packed, with a mean fiber diameter of 1.7 ± 0.47 µm SD. Likewise, a low dose of glyphosate induces an increase in collagen fiber diameter (1.43 ± 0.33 mm SD) compared with the control groups (1.03 ± 0.25 mm SD) (Figure 3B). A large effect size (>0.8) was reported between the analyzed groups.

### 2.3. Effects of Glyphosate on Carbohydrate Composition

**Figure 4:**
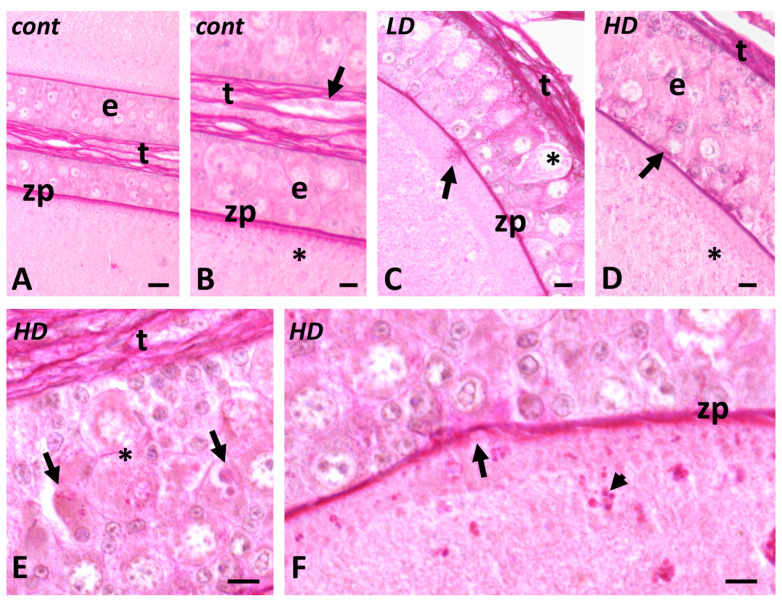
Effects of glyphosate (low LD and high HD doses) on carbohydrate distribution. PAS staining. (**A**,**B**) Stained zona pellucida (zp) and connective theca (t). Faintly stained epithelial cells (e) and oocyte cytoplasm (*). (**C**) Appearance of stained granules in follicle cells (arrow). Dense theca (t). (**D**) Release of PAS-positive granules (*) into the oocyte cytoplasm (arrow). Apoptotic cell (*). (**E**) Dense, poorly stained amorphic bodies (arrows); stained theca (t) and apoptotic pyriforms (*). (**F**) Disorganized zona pellucida (zp (arrow); PAS-positive granules (arrowhead) in the oocyte cortical cytoplasm. Scale bars: (**A**) 20 µm; (**B**–**F**) 10 µm.

In control follicles, PAS intensely stains the connective theca and the zona pellucida. Follicle cells’ cytoplasm is moderately stained as the oocyte cytoplasm (Figure 4A,B). After exposure to glyphosate, follicle cells’ cytoplasm is filled with small PAS-positive granular material (Figure 4C) that is released in the oocyte cortical cytoplasm via intercellular bridges (Figure 4D). At the higher dose, staining also reveals the presence of large, amorphic PAS-negative bodies (Figure 4E) and alterations to the zona pellucida become more frequent (Figure 4F).

**Figure 5:**
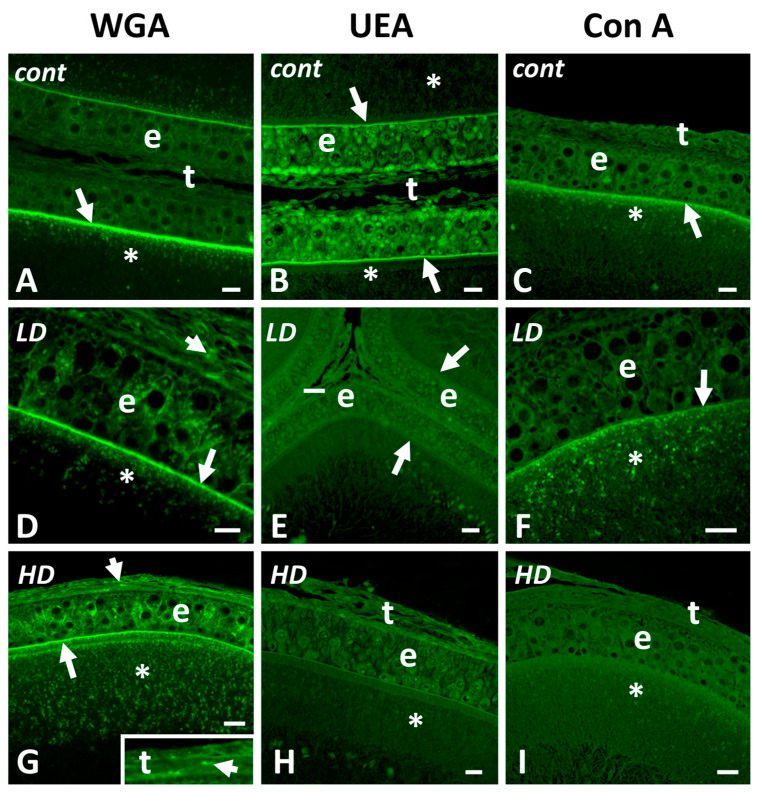
Effects of glyphosate (low LD and high HD doses) on carbohydrate distribution in ovarian follicles. Staining with fluorescent lectins. (**A**) Stained zona pellucida (arrow) and oocyte cortical granules (*); unstained theca (t) and epithelium (e). (**B**) Stained zona pellucida (arrows), follicular epithelium (e), and theca (t); unstained oocyte cortical cytoplasm (*). (**C**) Stained zona pellucida (arrow) and cortical granules (*); unstained epithelium (e). (**D**,**G**) Stained theca cells (t), zona pellucida (arrow), and cytoplasm of epithelial cells (e); note the increase in cortical granules (*). Inset: detail of theca with stained cells (arrowhead). (**E**,**H**) Unstained theca (t), epithelium (e), and oocyte cytoplasm (*). (**F**,**I**) Disappearance of staining on zona pellucida (arrow) and cortical granules (*). WGA stains N-Acetyl-glucosamine, UEA stains fucose, and Con A stains mannose. Scale bars: (**A–D; F–I**) 20 µm; (**E**) 40 µm.

In controls, the three lectins stain the zona pellucida; WGA (Figure 5A) and Con A (Figure 5C) also stain the cytoplasmic granules present in the oocyte cortex. UEA stains follicle cells’ cytoplasm, in particular that of small cells and thecal connectives (Figure 5B). 

After exposure to glyphosate, no matter the dose, UEA (Figure 5E,H) and Con A (Figure 5F,I) staining completely disappear on all follicular compartments. In contrast, a moderate WGA staining (Figure 5D,G) appears on pyriform cells’ cytoplasm and on occasional thecal cells (Figure 5G inset). Lectin also reveals a significant increase in the number of stained cortical granules (Figure 5D,G).

### 2.4. Effects of Glyphosate on the Presence and Localization of E-Caderin and PCNA

**Figure 6:**
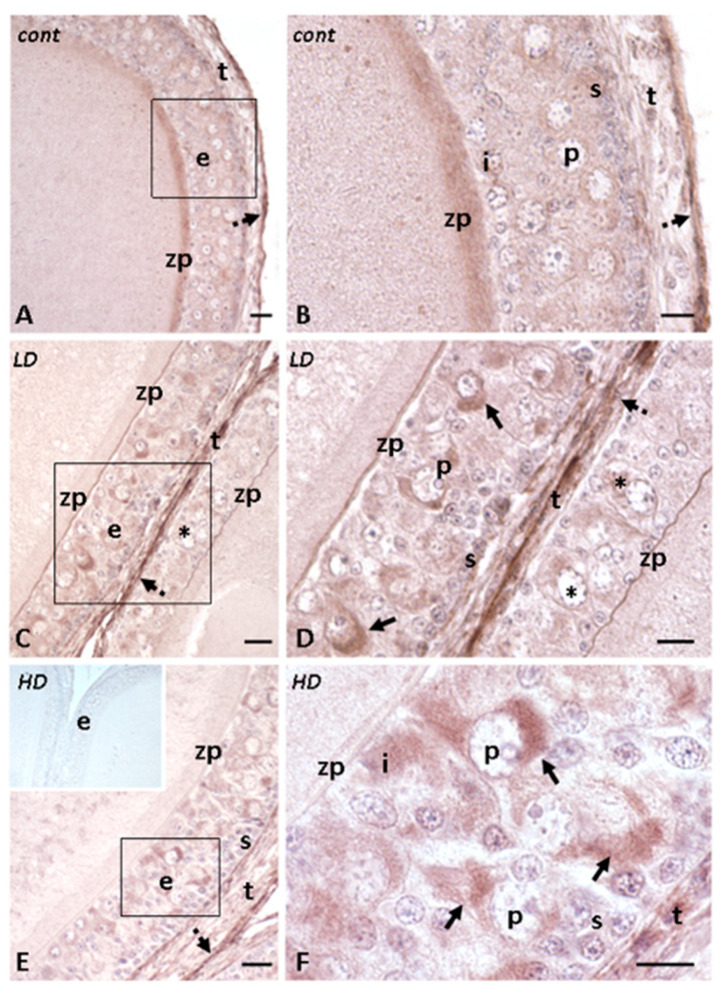
Immunohistochemical localization (brown areas) of E-cadherin in follicles of *P. siculus* treated with glyphosate (low LD and high HD doses). (**A**,**B**) Diffuse signal on the zona pellucida and follicular cells. Note the intense signal on theca externa (dotted arrows). (**C**,**D**) Intense signal (arrows) on the cytoplasm of occasional pyriform (P) cells and on theca (t). The epithelium (e) in a smaller oocyte is almost unstained and shows apoptotic pyriform cells (*). Zona pellucida (zp) is always stained. (**E**,**F**) Signal is on most pyriform (P), intermediate (I), and theca cells (t). E inset: negative control of the reaction. Scale bars: left images, 40 μm; right images, 20 μm.

In control follicles, a low and diffuse signal for E-cadherin is found on the zona pellucida and on the epithelium, while a moderately intense signal is on the external theca (Figure 6A,B). In animals treated with a low dose of glyphosate, an intense signal is present on the epithelia of larger follicles (1000 to 1400 µm diameter) in which E-cadherin concentrates in the cytoplasm of pyriform and theca cells (Figure 6C,D). At a higher dose, the signal on the zona pellucida reduces while the signal on epithelial cells increases (Figure 6E,F). In smaller follicles, no significant changes are observed (Figure 6C). 

**Figure 7:**
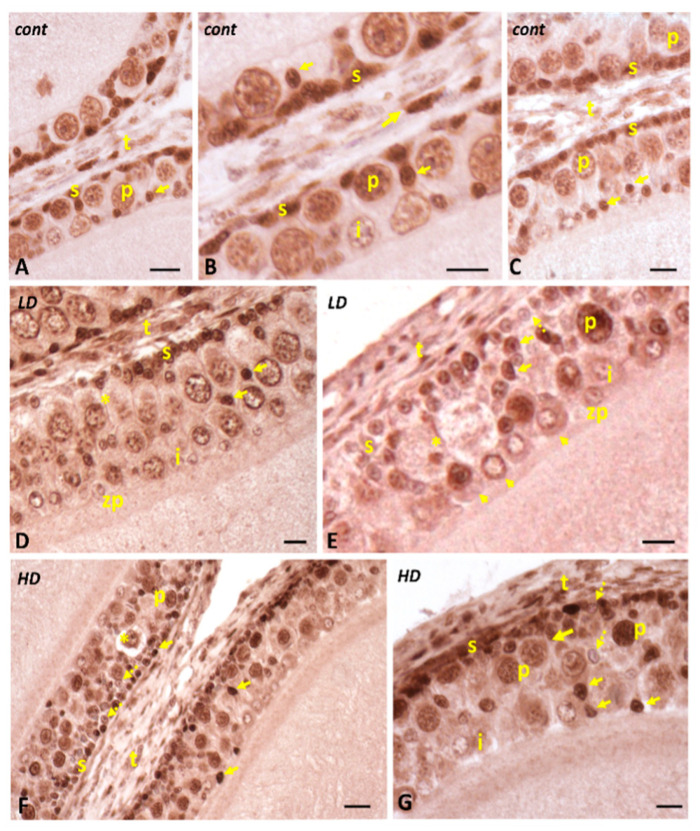
Immunohistochemical localization (brown areas) of PCNA in follicles of *P. siculus* treated with low (LD) or high (HD) doses of glyphosate. (**A**–**C**) Signal is evident on small (s) and theca cells’ (t) nuclei and on the nuclei of S cells migrating toward the zona pellucida (arrows). Intermediate (i) and pyriform (p) cell nuclei are moderately stained. (**D**) Intensely stained small (s) and theca (t) cell nuclei. Notice the stain on migrating S cells (arrows) and the presence of early apoptotic pyriform cells (*). (**E**) Stained small (s), pyriform (p), and theca (t) cell nuclei. Notice the stain on migrating S cells (arrows) and nuclei and/or cytoplasm of intermediate cells (i) (arrowheads). Occasional unstained S cells (dotted arrow) and apoptotic pyriforms (*). (**F**,**G**) Stained (arrows and unstained (dotted arrows) small cell nuclei. Notice the stain on migrating S cells (arrows), the occasionally unstained S cells (dotted arrows), and the presence of apoptotic pyriforms (*). Scale bars: 20 µm.

In controls, the immunohistochemical signal for PCNA antigen is present in the theca cells and within small stem cells, located immediately below the theca, as well as on small cells that migrate to the zona pellucida [28] and differentiate into intermediate cells (Figure 7A–C). In glyphosate-treated animals, in smaller follicles (150–1200 µm in diameter), the PCNA signal is found, as in controls, on the nuclei of small cells, located near the theca or in the epithelium (Figure 7D). In larger follicles (1200–1400 µm), those in which the small cells are organized in a typical bi-/trilayer, a diffuse, sometimes very intense signal is observed not only at the small cells, but also within the nuclei of pyriform and intermediate cells and or on the cytoplasm, while occasionally, the nuclei of small cells are not stained (Figure 7F,G). The nuclei of oocytes are always stained.

### 2.5. Effects of Glyphosate on the Presence and Localization of Alpha and Beta Estrogen Receptors

**Figure 8:**
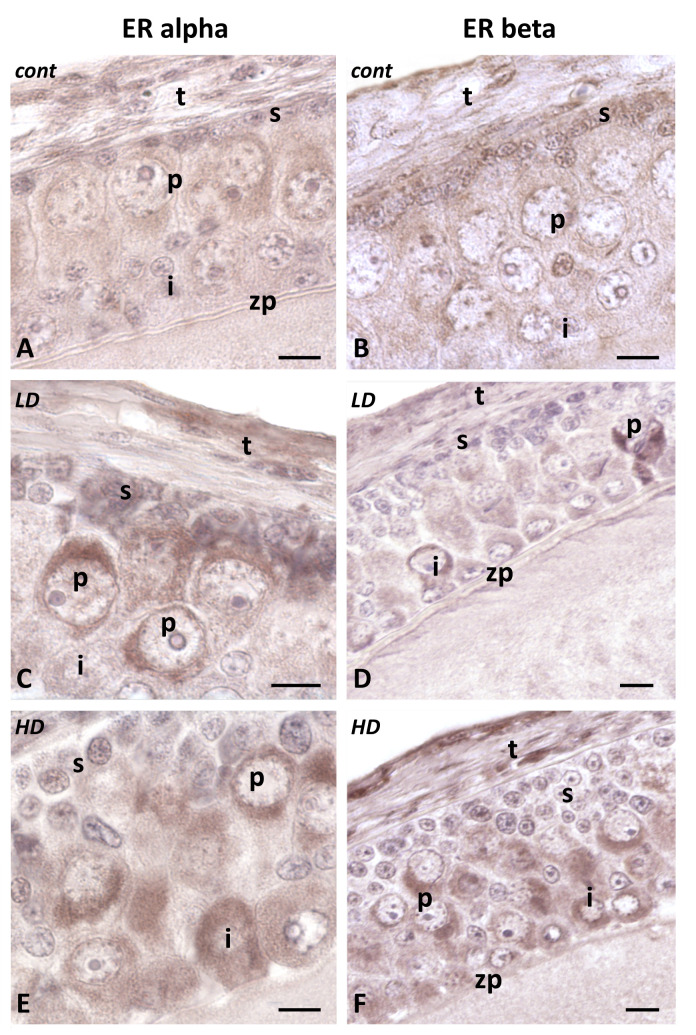
Immunohistochemical localization (brown areas) of ER α and ER β in follicles of *P. siculus* treated with low (LD) or high (HD) doses of glyphosate. (**A**,**B**) Weak signal on the epithelium. Small (s), intermediate (i), pyriform (p), and theca (t) cells. Zona pellucida (zp). (**C**,**D**) Appearance of an intense signal on the cytoplasm of occasional intermediate (i) and pyriform (p) cells. (**E**,**F**) Intensification of the signal on intermediate (i) and pyriform (p) cell cytoplasm. Stained theca cells (t), unstained small (s) cells, and zona pellucida (zp). Scale bars: 20 µm.

Glyphosate exposure causes a significant variation in both *ER-α* (Figure 8 left panels) and *ER-β* (Figure 8 right panels). In controls (Figure 8A,B), the signal is low and homogeneously distributed on the epithelium and the theca. The zona pellucida and the oocyte are completely unstained. The signal increases dose-dependently in animals treated with glyphosate. At a low dose (Figure 8C,D), a stain appears on the cytoplasm of occasional intermediate and pyriform cells; at a higher dose, the number of stained cells significantly increases and, occasionally, small and theca cells also become stained (Figure 8E,F).

## 3. Discussion

The results demonstrated that glyphosate, at both doses tested, profoundly interferes with ovarian function. It alters oocytes’ and follicles’ recruitment and follicular anatomy and, in the epithelium, increases estradiol receptors’ expression and anticipates physiological apoptosis. Such a plethora of effects, in addition to the alteration of the estrogenic system, could be related to the ability of glyphosate to mistakenly substitute glycine into peptides during protein synthesis [32]. This would cause direct and/or indirect alteration to proteins involved in energetics, oxidative stress, and mitochondrial function [33].

Glyphosate unquestionably interferes with the dynamic interplay between the follicular cells and their surrounding interfollicular ECM in the female reproductive system, inducing a production of a fibrotic-like connective tissue that exerts mechanical forces on neighboring cells. The most evident damage in Podarcis ovaries is observed in the theca, the collagen-rich compartment, enveloping the follicles, which become very thick and compact. Collagen accumulation has already been reported in the liver [24,34] and in the testis in Podarcis lizards [25,35,36] and rats [37]. Therefore, thecal cells are markedly compressed among fibers and apparently reduced in number. They, however, would maintain their function [38], as the synthesis of androgens is essential for estradiol production by granulosa cells [39].

It cannot be excluded, however, that thecal compaction would exert mechanical effects. Compression of the hilar blood vessels would alter the oxygen supply to the developing follicles. In addition, tensile strength due to the theca thickening could represent an obstacle at the time of ovulation, when collagens around the follicle are remodeled, via the release of lytic substances [40,41]. Site-directed degradation mechanisms might be insufficient to cope with such a dense sheath, thus impairing/retarding follicular rupture. This unusual collagen deposition could also indicate the onset of ovarian tissue fibrosis, which could lead to infertility. It is significant that theca thickening is accompanied by an increase in N-Ac-glucosamine, a disaccharide present in hyaluronan, a linear high-molecular-weight glycosaminoglycan typical of the theca interna [42]. Further changes are demonstrated by UEA staining. Thecal proteoglycans play a fundamental role in generating swelling pressure, thus promoting solvent influx into the follicles [43]. Altered glycation could thus have severe effects and, in Podarcis follicular epithelium, it might explain the patches of shrunken cells observed in glyphosate-treated animals.

Marked effects are also registered at the level of the follicular epithelium. The most striking is the induction of apoptosis at a stage, the early previtellogenesis, in which apoptosis should be completely absent. Pyriforms are nurse cells [28], committed to apoptosis [44], but the process begins at the end of previtellogenesis when follicles reach about 1500 µm in diameter and pyriform cells expose GnRH receptors [45]. In addition, regression is asynchronous and only a few apoptotic cells are visible at a time in sections obtained from control animals [46].

Several possible mechanisms may account for the anticipated degeneration of pyriform cells. The first is supported by literature and suggests that glyphosate induced oxidative stress [47]. Pyriform cells are very rich in mitochondria owing to their nurse function [28] and, indeed, would represent an easy target for the herbicide. Mitochondrial effects have been already reported, in Danio [48] and mice [2], for example.

A second possibility is suggested by the observed changes in carbohydrate presence and distribution: PAS-positive granules are formed, and a cytoplasmic increase in glcNAc and a decrease in fucose are observed. Glyphosate interference with carbohydrates is well known; it alters pathways involved in fucose synthesis, transport, and attachment to glycoproteins [49,50] as well as in the production of uridine diphosphate N-acetylglucosamine (UDP-GlcNAc). This nucleotide sugar is a precursor to many other derivative sugars that are incorporated into the heparan sulphate and chondroitin sulphate chains in glycosylated proteins [49]. Alteration in extracellular matrix glycoconjugates starts apoptosis in granulosa cells [51], and heparan sulphate and heparan sulphate proteoglycans control proliferation and differentiation [52]. In Podarcis follicles, the epithelial cells are thus the perfect target as the three processes coexist. Proliferation occurs in small stem cells; differentiation transforms small cells into large nurse pyriform cells [53] and apoptosis eliminates them in preparation for vitellogenesis [46]. Furthermore, the reduction of fucose at the level of the zona pellucida, as demonstrated by lectin imaging, could also impair fertilization processes, as it is known in the literature that this sugar is critical for the interaction of sperm with the gelatinous layer and the subsequent acrosomal reaction [54].

The same dysregulation that anticipated apoptosis did not increase the proliferation rate in small cells. The results obtained with PCNA, in fact, accord with the evidence that small cells proliferate during early previtellogenesis [55], but not with the evidence that glyphosate stimulates proliferation [38]. The difference with respect to the reported literature may reside in the fact that follicular epithelium in *Podarcis siculus* is a highly specialized, timed structure that undergoes an environmentally and genetically programmed series of events [56]. Unexpected is the signal found on pyriform and intermediate cells, for which no explanations are available. However, it is intriguing that a report demonstrates that PCNA plays an important role in apoptosis-mediated oocyte loss during primordial follicle formation in mouse ovaries [57]. This is also a genetically controlled process and a parallel with pyriform cell degeneration is suggestive. Unfortunately, as far as we are aware, there is no evidence in the literature on the involvement of PCNA in granulosa cell degeneration. 

Proliferation, therefore, may not account for the epithelial thickening observed in several follicles, but the thickening could be determined by the difficulty of lateral sliding of the cells owing to the excess of external collagen. As a result, the cells slide more easily downwards in the direction of the zona pellucida, which appears undulating in several places owing to the mechanical forces exerted by the follicular cells. Similar alterations have been described after cadmium exposure [58], but, in that case, a loss of cell contact was also observed. In glyphosate-treated follicles, granulosa cells remain in strict contact with each other, evidence contrasting with the accumulation of E-cadherin in their cytoplasm.

Another interesting result is the decreased signal for E-cadherin at the level of the zona pellucida, evidence indicating that contact between follicle cells and the oocyte is altered. The zona pellucida also shows morphological alterations and a loss in fucose and mannose, two sugars fundamental for fertilization [59]. It is easy to conclude that glyphosate induced structural and functional impairment of the zona pellucida. This, however, is not the only damage exerted on the oocyte. The poor condition of the cytoplasm indicates profound interference with the cytoskeleton, with microtubules [60] and actin [61], with potential variations in membrane rigidity.

Further evidence of damage comes from the observed dose-dependent increase in estrogen receptors, alpha and beta, in the pyriform cell cytoplasm. The literature suggests that glyphosate has estrogenic properties [24,25,62] and that effects are exerted via inhibition of aromatase enzyme activity [63] and via estrogen receptors’ activation [64], and how natural estrogens might have increased gene expression of the same ERs was recently demonstrated in the testis of *P. siculus* [25]. However, opposite evidence has also been collected [65], thus the endocrine-disruptive capability of glyphosate remains uncertain.

In *P. siculus* follicle cells, the ER receptors increase, dose-dependently, after exposure, but they remain localized in the cytoplasm, thus suggesting that the observed damages are linked to a ligand-independent mechanism more than a conventional, genomic, endocrine-disrupting effect [66]. Glyphosate indeed changes the expression of GnRH, LHR, FSHR, 3β-HSD, and Cyp19a1 genes in mice [2], and in lambs, it decreases mRNA expression of FSHR and GDF9 in the ovary, but not in the uterus [67]. It is thus quite evident that tissue-specific effects should be expected and that more studies are needed to clarify the mechanism of action and distinguish between causes and effects. It should be specified that the experiments in *P. siculus* were conducted in fall, a period in which the ovary is in a recrudescence phase [56], but not all the activities have been resumed. In this period, in fact, the ovary is responsive to exogenous FSH administration and to changes in photoperiod, but only if these anticipate the regime of the incoming season [68]. The accumulation of ER in the cytoplasm thus might have had no consequences only because there was no circulating estradiol in the plasma [69]. Glyphosate administered in another phase of the reproductive cycle might have had completely different consequences.

Glyphosate, however, stimulated the ovary, also in fall; it induced the recruitment of early diplotene oocytes from the pool of zygotene oocytes (that almost disappeared from the germinal beds) and induced the recruitment of extra primary follicles. Significantly, recruitment is not accompanied by oogonial proliferation or the appearance of new leptotene oocytes; therefore, more than true recruitment, it appears that glyphosate has anticipated differentiation, an effect parallel to the anticipated apoptosis in the follicular epithelium. A check for an anticipated exposure of GnRH receptors might prove that this is the case [45].

It is impossible to know if the newly recruited oocytes would have been ovulated because, in Podarcis, the maximum number of eggs is strictly controlled and depends on female size more than on environmental cues [68,70]. It is, however, quite interesting that no atresia was observed, though treatment lasted for 3 weeks, and glyphosate induces follicular granulosa degeneration [6]. In Podarcis, follicle selection occurs in early diplotene, before the formation of the primordial follicle [71], a convenient strategy for a small species that produces large and telolecithal eggs.

In conclusion, exposure to glyphosate results in structural and functional damage. These injuries, combined with those found on the morpho-physiology of the testis in male specimens [25], suggest serious damage to the reproductive fitness of this species. Further studies will be needed to verify this scenario, analyzing the effects of glyphosate in the mating period and, if fertilization occurs, on the embryos.

## 4. Materials and Methods

### 4.1. Animals

*Podarcis siculus* females were captured in late October in the outskirts of Naples (Campania, Italy), maintained in soil-filled terrariums under natural temperature and photoperiod (20 °C, 10 h daylight), and fed with insect larvae. After 7 days of acclimatization, the animals were randomly assigned to three groups (*n* = 8): groups 1 and 2 were exposed to the active ingredient glyphosate (Gly) at doses of 0.05 (low dose, LD) and 0.5 (high dose, HD) μg/kg body weight, respectively; group 3 were left as control animals. Gly was administered by pipetting the corresponding aqueous solution (50 µL) into the mouth, three times a week, for 3 weeks; control animals received pure water. At the end of the treatments, the animals were killed by decapitation after anesthesia with ketamine hydrochloride (Parke-Davis, Berlin, Germany), at 325 μg/g body weight; ovaries were excised and processed for histological and immunocytochemical analyses.

### 4.2. Histological Analysis

Ovaries were fixed in Bouin’s solution, dehydrated in a graded series of ethanol, clarified in xylene, and embedded in paraffin. Sections (7 μm thick) were dewaxed, rehydrated, and stained with Mallory’s trichrome [72] to show the general morphology. To highlight glycogen and glycoproteins, sections were stained with the Periodic acid Schiff (PAS). Sugar residues were characterized by staining with FITC-conjugated lectins (Vector Laboratories Inc., Newark, CA, USA; 2 mg/mL) [73]. Concanavalin A (Con A) was used to reveal mannose, UEA to reveal fucose, and WGA to reveal N-acetyl-glucosamine (glcNAc). Sections were covered with 1 μL of lectin diluted in 19 μL of PBS, incubated in a dark moist chamber at room temperature for 15 min, rinsed with PBS, and observed under a UV microscope (excitation maximum at 495 nm and emission maximum at 515 nm). Negative controls, prepared by incubating slides with the lectin and the specific competing sugar or by omitting the lectin, were always unstained. Labeling was defined as positive or negative by two independent observers [74]. 

Sections were also stained with Picrosirius red to show collagen; polarized light images were acquired with a polarized light microscope. To quantitatively determine the proportion of mature (red) and immature (green) collagen fibers, randomly selected images were resolved into their hue, saturation, and brightness (HSB) components using the “color threshold” function of ImageJ® software (version 1.53t). The hue component was retained, and a histogram of hue frequency was obtained from the resolved 8-bit hue images (containing 256 colors). The following hue definitions were used: red 0–51 and green 52–120 [75,76]. Collagen fiber diameters in thecal layers were measured on randomly selected areas in 10 regions of interest (ROIs) of histological sections. The line selection tool of Image J^®^ software (version 1.53t) was used and the scale bar was reported on each image for calibration. The results were analyzed for significance by the Student’s *t*-test. Statistically significant differences among groups were tested by analysis of variance (ANOVA) or the Kruskal–Wallis test. Data are presented as average ± SD and *p*-values <0.05 were considered statistically significant. Furthermore, Cohen’s d was used to calculate the effect size through a standardized mean difference. Cohen’s d = (x1 − x2)/s, where x1 and x2 are the sample means of group 1 and group 2, respectively, and s is the standard deviation of the population from which the two groups were taken. All of the stained sections were observed using a Zeiss Axioskop microscope and the images were acquired using Axiovision 4.7 Software (Zeiss, Oberkochen, Germany) [77].

### 4.3. Immunohistochemistry

Sections mounted on polylysine glass slides were treated with 10 mM citrate buffer pH 6.0 to unmask the antigens and incubated in 2.5% H_2_O_2_ in methanol to block the endogenous peroxidase [78,79]. Non-specific signals were reduced by incubation in normal goat serum (Pierce, Rockford, IL, USA) for 1 h at room temperature. The slides were incubated overnight at 4 °C with the following primary antibodies diluted in normal goat serum: rabbit anti-human E-cadherin (1:300); rabbit anti-human PCNA (1:300); rabbit anti-human ERα (1:200); and rabbit anti-ERβ (1:200). All antibodies were from Santa Cruz Biotechnology (Santa Cruz, CA, USA), except anti-human PCNA, which was from Elabscience (Houston, TX, USA). The next day, the sections were incubated with HRP-conjugated goat-anti-rabbit or mouse secondary antibody diluted 1:200 in normal goat serum for 1 h at room temperature, and then incubated with avidin–biotin–peroxidase complex (ABC immune peroxidase kit, Pierce) for 1 h at room temperature. Finally, the sections were stained with diaminobenzidine (DAB) as chromogen and counterstained with Meyer’s hematoxylin. Negative controls were obtained by omitting incubation with the primary antibody. The immunohistochemical signal was observed with a Zeiss Axioskop microscope and images were acquired with Axiovision 4.7 software (Zeiss, Oberkochen, Germany).

## Data Availability

The authors confirm that the data supporting the findings of this study are available within the article.
